# Case Report: ^18^F-FDG PET/CT and Laparoscopic Nephron Sparing Surgery in the Management of Bleeding From Renal Metastases of Choriocarcinoma

**DOI:** 10.3389/fonc.2022.829190

**Published:** 2022-04-14

**Authors:** Yuancheng Du, Xueping Zhang, Shengyang Sun, Meihong Sun, Dongyu Yang, Xinyuan Yu, Kehao Li, Jie Ma, Yongxiang Li, Jinming Ge, Changqing Liu, Liang Qiao

**Affiliations:** ^1^ Department of Urology, Weifang Medical University, Weifang, China; ^2^ Department of Urology, Weifang People’s Hospital, Weifang, China; ^3^ Obstetrics and Gynecology Department, Zichuan District Hospital, Zibo, China; ^4^ Department of Hematology, Linyi People’s Hospital, Linyi, China; ^5^ Department of Radiology, Weifang People’s Hospital, Weifang, China

**Keywords:** choriocarcinoma, spontaneous renal hemorrhage, PET/CT, renal metastasis, laparoscopic nephron sparing surgery

## Abstract

Choriocarcinoma is a cancer that usually occurs in the uterus during pregnancy. Although choriocarcinoma with renal metastasis and spontaneous renal hemorrhage is very rare, it can occur. We describe a rare case of metastatic choriocarcinoma, wherein the patient presented with acute abdominal pain due to a subcapsular hematoma secondary to a bleeding renal metastasis. We performed a laparoscopic nephron sparing surgery to remove the tumor and control the bleeding. A retrospective analysis revealed that metastasis was detected on ^18^F-fluorodeoxyglucose PET/CT, but not on CT alone. To our knowledge, a case of choriocarcinoma with such symptoms and treatment has not been described in recent literature. Our case illustrates that acute bleeding from a renal metastasis can be effectively managed by laparoscopic nephron sparing surgery. It also demonstrates the advantage ^18^F-FDG PET/CT may have in the evaluation of metastatic choriocarcinoma.

## Introduction

When spontaneous renal hemorrhage occurs, the first considerations for urologists are the possible presence of a tumor and the need for surgical treatment. However, when a mass is not found on preoperative computed tomography (CT), it may affect the choice of conservative management. Choriocarcinoma is a highly malignant trophoblastic neoplasm, which is characterized by trophoblastic cells losing their original villi or hydatidiform mole structure, infiltrating into the myometrium, causing serious local damage, and metastasizing to other structures. Most choriocarcinomas secondary to normal or abnormal pregnancy occur in women of childbearing age. Distant metastasis of choriocarcinoma is closely related to the location of metastasis. Renal metastasis with spontaneous renal bleeding is very rare (1, 2). In patients with metastatic choriocarcinoma, ^18^F-fluorodeoxyglucose positron emission tomography (^18^F-FDG PET) can identify occult diseases when ultrasonography, CT, and magnetic resonance imaging (MRI) are not clear. Although there are some reports on renal metastasis of choriocarcinoma with spontaneous renal hemorrhage in existing literature, few authors have discussed the manifestations of renal choriocarcinoma on PET/CT in detail. We report a case of laparoscopic treatment of a spontaneous renal hemorrhage secondary to a choriocarcinoma renal metastasis along with the PET/CT findings, and summarize the experience through a literature review to guide correct preoperative diagnosis and improve surgical results.

## Case Description

A 37-year-old Chinese woman presented to our hospital with hemoptysis. Enhanced chest CT showed a solid-cystic mass in the right lung. Her past medical history indicated that she underwent a cesarean section in 2008. Menstrual history suggested irregular menstruation, and childbearing history included three pregnancies, one childbirth and two miscarriages.

**Figure 1:**
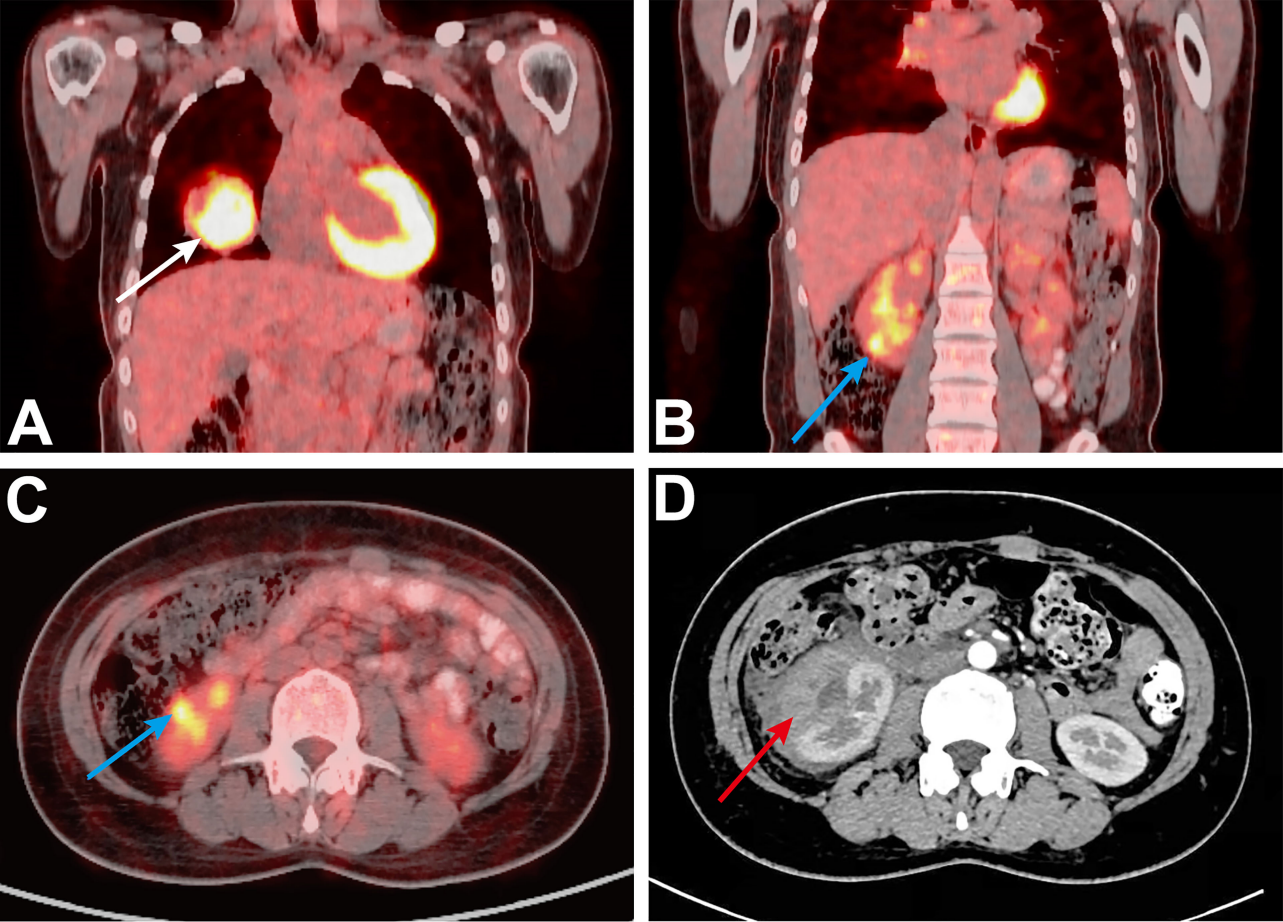
**(A)** Initial PET/CT, showing the right lung mass (solid white arrow). **(B, C)** Initial PET/CT, showing the mass of the lower pole of the right kidney (SUV_max_ = 8.7, solid blue arrow). **(D)** Preoperative CT shows a large subcapsular hematoma (solid red arrow).

**Figure 2:**
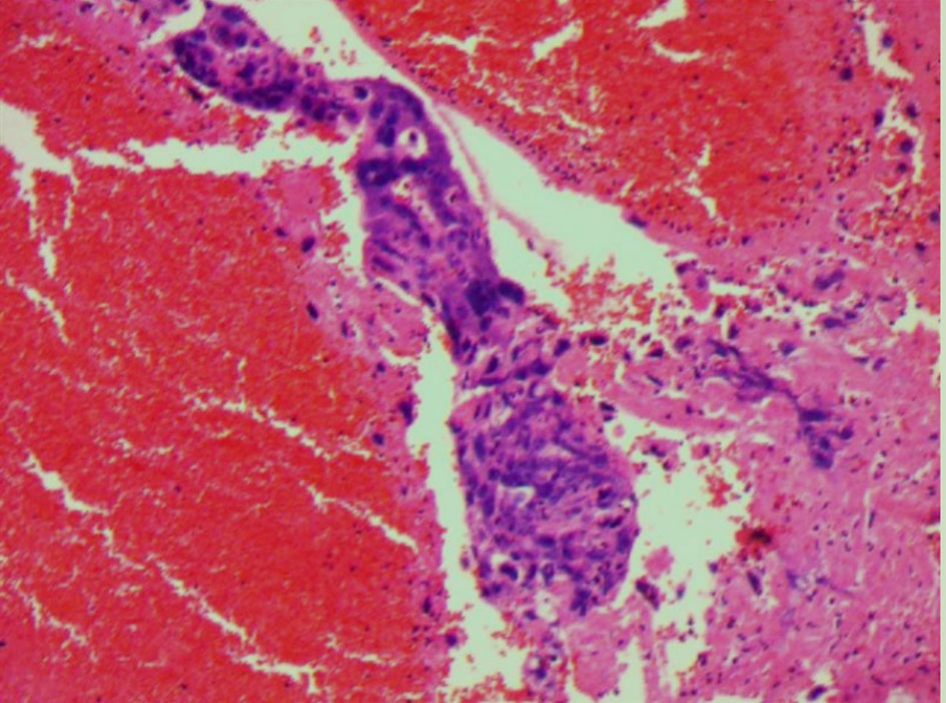
The final histology shows metastatic choriocarcinoma of the kidney.

**Figure 3:**
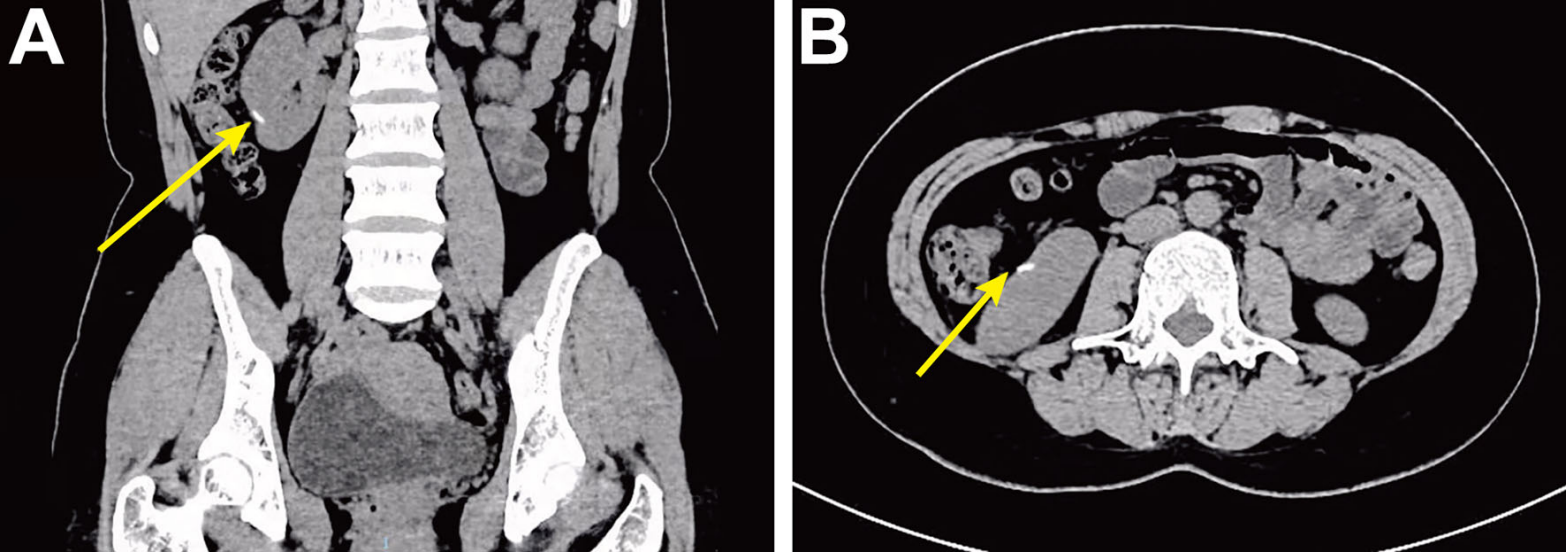
Postoperative follow-up high-density CT shows surgical area marked by hemolok (solid yellow arrow). **(A)** Coronal CT. **(B)** Axial CT.

On the first day of hospitalization, the patient underwent a fine-needle biopsy of the right lung. Upon pathologic examination with immunohistochemistry, lung metastasis of choriocarcinoma was first considered. Immunohistochemistry detected human chorionic gonadotropin (HCG), while β-HCG levels obtained were 279,064.00 mIU/ml. ^18^F-FDG PET/CT examination revealed a mass in the middle lobe of the right lung, with unevenly increased metabolic uptake (in line with the signs of lung carcinoma), with an approximate size of 5.3 cm × 5.8 cm and SUV_max_ of 18.3 (**Figure 1A**). The patient suddenly experienced acute pain in the right waist and abdomen. Ultrasound and enhanced abdominal CT showed irregular high-density shadows under the capsule of the right kidney, uneven density, and compressed right kidney parenchyma. Spontaneous renal hemorrhage and renal subcapsular hematoma were considered (**Figure 1D**). Laboratory examination indicated a red blood cell count of 2.73×10^12^/L and a hemoglobin level of 82 g/L. After conservative treatment, the clinical status of the patient was marked by a rapid deterioration: hemodynamic instability, worsening flank pain, and continuous decrease of the red blood cell count. Urologists planned emergency laparoscopic exploration. During the operation, many blood clots under the capsule were found. Upon their removal, a tumor with an approximate diameter of 1.5 cm was found on the ventral side of the kidney. Since angiomyolipoma and renal cell carcinoma are the most common causes of spontaneous renal hemorrhage, these were first considered. However, we could not exclude a diagnosis of a malignant tumor because of the lesions in the lungs. Therefore, a nephron sparing surgery was sufficient to control the bleeding, resolve symptoms, and provide surgical specimens. Postoperative pathology showed that heterozygous cell nests were found in the clot, which was consistent with choriocarcinoma combined with HCG (+) on immunohistochemistry (**Figure 2**). After surgical treatment, the patient was referred to the oncology department for chemotherapy, which included regimens of etoposide, methotrexate, actinomycin D, cyclophosphamide, and vincristine (EMA-CO) and paclitaxel-cisplatin/paclitaxel-etoposide (TP/TE). During the treatment period, the β-HCG level was as low as 3.67 mIU/ml. Clinical follow-up with chest, abdominal, and pelvic CT scan at 6 and 9 months showed lung disease reduction without signs of recurrence in the kidney (**Figures 3A, B**). Nevertheless, the patient developed symptoms of brain metastases 1 year later. Multiple metastases in the brain were observed on craniocerebral MRI. After receiving brain radiotherapy, due to obvious side effects and continued disease progression, the patient chose to stop active treatment and died within 1 year of diagnosis.

## Discussion

This patient was diagnosed with stage IV choriocarcinoma with renal metastasis, spontaneous renal hemorrhage, and lung and brain metastases. Intracranial hypertension was the direct cause of death.

Gestational choriocarcinoma is a pure epithelial malignancy, composed of neoplastic intermediate trophoblasts, cytotrophoblasts, and syncytiotrophoblasts without chorionic villi. Central necrosis and hemorrhage are frequently observed in choriocarcinoma. Patients with gestational choriocarcinomas tend to develop early systemic metastases (1). The most common sites of metastasis are the lung, vagina, liver, digestive tract, and brain. Renal metastasis is exceedingly rare (1–3). The International Association for the Study of Trophoblastic Diseases (ISSTD) states that the incidence of renal metastasis of choriocarcinoma is 1% (4). The common feature of symptoms in all metastatic sites is local bleeding. Hemoptysis can occur in cases of lung metastasis, accompanied by chest tightness, gastrointestinal metastasis, and gastrointestinal bleeding. In this case, the symptom of renal metastasis of choriocarcinoma is spontaneous renal bleeding, which is consistent with the growth characteristics of trophoblasts. In a review of 448 patients with choriocarcinoma by Wang et al. (5), 15 patients (3%) had clinical symptoms and signs of renal metastasis (e.g., hematuria and renal pain) and one patient (0.2%) had a renal subcapsular hematoma.

Our case shows that in a specific clinical situation, non-gynecological symptoms may become the first symptom of choriocarcinoma. The common clinical manifestation of choriocarcinoma is abnormal vaginal bleeding, but there are also many patients in whom the primary tumor resolves and only present with the symptoms of metastases. In most of the reported cases, there is a recurrent diagnostic problem: most cases were misdiagnosed with renal colic or renal tumors prior to histology or post-mortem examination. Some researchers recommend screening of HCG levels to exclude the possibility of choriocarcinoma metastasis in young women with hematuria and renal masses who are suspected to have renal cancer (6). Shinoda et al. (7) suggested that patients with extremely high β-HCG levels (>1000 mIU/ml) can be diagnosed with choriocarcinoma even before the pathology results are obtained. Our case supports this because the initial β-HCG level in our patient was much higher than this level, indicating the importance of the serum β-HCG level in the preoperative examination.

There are limited data on the use of PET/CT in the evaluation of metastatic choriocarcinoma. Sironi et al. (8) and other studies on choriocarcinoma cases demonstrated that PET/CT has a higher application value than CT alone in assessing metastases. PET/CT could effectively detect occult choriocarcinoma lesions that cannot be detected using traditional imaging, i.e., MRI or CT. In this case, after the diagnosis of lung metastasis of choriocarcinoma, spontaneous renal hemorrhage occurred during treatment. Abdominal enhanced CT was negative, but in the retrospective analysis of FDG PET/CT images (**Figures 1B, C**), we found a lesion that was postoperatively proven to be a tumor. Thus, ^18^F-FDG PET/CT may have an advantage in the evaluation of metastasis choriocarcinoma.

The laparoscopic approach is effective, less invasive, and allows faster recovery than the open surgical approach. Renal function was not affected by the choice of nephron sparing surgery rather than radical nephrectomy, and the patient became more tolerant to postoperative chemotherapy. In a similar case, Li et al. (9) used the open surgical approach and partial nephrectomy to control the bleeding. In the cases of Vijay et al. (2) and Lal et al. (10), spontaneous renal hemorrhage caused by renal metastasis of choriocarcinoma was managed with angioembolization of the renal masses with subsequent angiography embolization of the renal lesions. Additionally, Scarcella et al. (11) reported a case of germ cell tumor renal metastasis. After spontaneous renal hemorrhage of a lesion of considerable volume, open radical nephrectomy was performed to control the bleeding. No hemorrhage or metastasis was observed in the postoperative follow-up period.

The limitations of this paper are related to the existing literature on this area, which are mainly composed of single-center studies with small sample sizes. A multi-center study with a larger sample size is required.

In conclusion, compared with our case, laparoscopic nephron sparing surgery can effectively control acute hemorrhage when spontaneous renal hemorrhage caused by a small-volume metastatic tumor occurs. It may also have more advantages in postoperative recovery, reduced tumor burden, and provision of histological biopsies. However, this modality is limited by the location and size of the tumor. Therefore, for patients with contraindications to laparoscopic nephron sparing surgery, angiography embolization and open radical nephrectomy are alternative therapies. It remains imperative that surgical methods are selected according to the patient’s situation.

## Data Availability Statement

The original contributions presented in the study are included in the article/supplementary material. Further inquiries can be directed to the corresponding author.

## Ethics Statement

Ethical review and approval was not required for the study on human participants in accordance with the local legislation and institutional requirements. The patients/participants provided their written informed consent to participate in this study. Written informed consent was obtained from the individual(s) for the publication of any potentially identifiable images or data included in this article.

## Author Contributions

YD, XZ, and LQ have made substantial contributions to the conception and design and have been involved in drafting the paper. SS, MS, KL, JM, and JG have performed data acquisition. YD, DY, and XY revised the manuscript. YL and CL performed the review of literature. All authors contributed to the article and approved the submitted version.

## Conflict of Interest

The authors declare that the research was conducted in the absence of any commercial or financial relationships that could be construed as a potential conflict of interest.

## Publisher’s Note

All claims expressed in this article are solely those of the authors and do not necessarily represent those of their affiliated organizations, or those of the publisher, the editors and the reviewers. Any product that may be evaluated in this article, or claim that may be made by its manufacturer, is not guaranteed or endorsed by the publisher.
